# Fluid Biomarkers of Disease Burden and Cognitive Dysfunction in Progressive Supranuclear Palsy

**DOI:** 10.1002/acn3.70327

**Published:** 2026-01-28

**Authors:** Roxane Dilcher, Charles B. Malpas, Stuart J. McDonald, William T. O’Brien, Craig Despott, Kelly L. Bertram, Matthew P. Pase, Lukas Frontzkowski, Meng Law, Terence J. O’Brien, Lucy Vivash

**Affiliations:** ^1^ Department of Neurosciences School of Translational Medicine, Monash University Melbourne Victoria Australia; ^2^ Department of Medicine Royal Melbourne Hospital, The University of Melbourne Melbourne Victoria Australia; ^3^ Melbourne School of Psychological Sciences The University of Melbourne Melbourne Victoria Australia; ^4^ Department of Neurology Alfred Hospital Melbourne Victoria Australia; ^5^ Department of Nuclear Medicine Alfred Health Melbourne Victoria Australia; ^6^ School of Psychological Sciences, Turner Institute for Brain and Mental Health Monash University Clayton Victoria Australia; ^7^ Harvard T.H. Chan School of Public Health Boston Massachusetts USA; ^8^ Institute for Stroke and Dementia Research, Klinikum der Universität München, Ludwig‐Maximilians‐Universität LMU Munich Germany; ^9^ Department of Radiology Alfred Health Melbourne Victoria Australia

**Keywords:** biomarkers, progressive supranuclear palsy, tau, tau‐PET

## Abstract

**Objective:**

Identifying objective biomarkers for progressive supranuclear palsy (PSP) is crucial to improving diagnosis and establishing clinical trial and treatment endpoints. This study evaluated fluid biomarkers in PSP versus controls and their associations with regional ^18^F‐PI‐2620 tau‐PET, clinical, and cognitive outcomes.

**Methods:**

Twenty‐four PSP patients and 11 age‐ and sex‐matched control subjects underwent Cerebrospinal fluid (CSF) and plasma assays of neurofilament light chain (NfL), glial fibrillary acidic protein (GFAP), and total tau (t‐tau) using SIMOA. Ratios (NfL/t‐tau, GFAP/t‐tau, GFAP/NfL) were calculated. Tau burden was quantified using PI‐2620 distribution volume ratio (DVR). Group comparisons and associations with tau‐PET, MRI, and cognition were tested using Wilcoxon tests, ROC analyses, and age‐ and sex‐adjusted linear models.

**Results:**

PSP patients showed elevated NfL, GFAP levels, and higher NfL/t‐tau and GFAP/t‐tau ratios in CSF and plasma. CSF NfL/t‐tau best discriminated PSP from control subjects (AUC = 0.99, 95% CI [0.97–1.00]; optimal cut‐off: > 10), followed by CSF GFAP/t‐tau (AUC = 0.89, 95% CI [0.76–1.00]; > 104) and plasma NfL/t‐tau (AUC = 0.80, 95% CI [0.63–0.96]; 5.1). Plasma NfL/t‐tau correlated with tau‐PET DVR in the putamen (*β* = 0.63; 95% CI [0.15–1.11]; *p* < 0.01) and pallidum (*β* = 0.54, 95% CI [0.07–1.02]; *p* < 0.01) and predicted disease severity (*β* = 0.61, 95% CI [0.19, 1.04]; *p* = 0.007) and processing speed (*β* = 0.66, 95% CI [0.22, 1.10]; *p* = 0.006), explaining 35% and 33% of variance, respectively. Frontal MRI volume modestly improved prediction of processing speed (Δ*R* ^2^adj = 0.23, *p* = 0.01), whereas tau‐PET did not.

**Interpretation:**

Plasma NfL/t‐tau correlates with regional tau, disease severity, and cognition. Fluid biomarkers, complemented by PET and MRI, may support multimodal PSP diagnosis, monitoring, and trial stratification.

**Trial Registration:**

Australian New Zealand Clinical Trials Registry: ACTRN12620001254987

## 1 Introduction

Tauopathies are neurodegenerative diseases characterized by the pathological accumulation of tau protein aggregates in the brain, leading to distinct patterns of neurodegeneration and symptom progression [1, 2, 3, 4]. Progressive supranuclear palsy (PSP) is a primary tauopathy, marked by midbrain atrophy and tau deposition in neurons and glial cells, predominantly in the brainstem and basal ganglia [5]. Clinically, PSP presents as an atypical parkinsonian syndrome with gait instability, frequent falls, oculomotor, and executive impairments [6]. Diagnosis currently relies on clinical criteria [7], and can only be definitively confirmed post‐mortem, with substantial delays in diagnosis and trial enrolment still common. Biomarkers are not intended to replace clinical judgment but to provide earlier, biology‐based evidence of disease to improve patient stratification, monitoring, and timing of therapeutic intervention, and thereby facilitate clinical trial design.

Neuroimaging has provided important insights into PSP neuropathology. MRI‐based measures of regional brain atrophy are reliable markers of neurodegeneration in PSP [8, 9, 10], while tau‐PET tracers, such as ^18^F‐PI‐2620, have shown promise in detecting regional tau pathology in PSP, corticobasal syndrome, and Alzheimer's disease. In vivo studies demonstrate elevated tau binding in the basal ganglia in PSP, which differs from the cortical tau distribution typically seen in Alzheimer's disease [11, 12].

Fluid biomarkers have also gained attention as potential tools for diagnosis and disease monitoring. Neurofilament light chain (NfL), a marker of axonal injury or degeneration, and glial fibrillary acidic protein (GFAP), a marker of astrocytic activation, are higher in cerebrospinal fluid (CSF) and peripheral blood in individuals with PSP compared with healthy controls [13, 14, 15, 16, 17]. Elevated NfL in PSP reflects more advanced disease, associating with greater clinical and cognitive impairment, and a poorer prognosis [15, 18, 19, 20]. By contrast, total tau (t‐tau), which is thought to reflect neuronal/axonal injury or degeneration and potentially tau pathology, is generally low in PSP [21, 22, 23], reflecting pathological differences compared with Alzheimer's disease. Although these markers may provide distinct biological insights into regional and temporal patterns of protein changes, their diagnostic specificity remains limited when used in isolation. Increasing evidence suggests that combining analytes into biomarker ratios may better capture disease‐relevant processes. Prior studies indicate that ratios outperform single markers by integrating disease‐specific pathology with general neurodegenerative burden [24, 25, 26]. In this context, the NfL/t‐tau ratio may reflect the imbalance between progressive neuroaxonal damage and relatively low soluble tau levels, a characteristic feature of PSP compared to Alzheimer's disease. Combining NfL, GFAP, and t‐tau into biomarker ratios may better capture the interplay between axonal injury, astrocyte pathology, and tau dysfunction, while improving diagnostic and prognostic utility.

Integrating fluid biomarkers with tau‐PET and MRI enables assessment of both systemic biological alterations and the spatial distribution of tau pathology and structural brain changes. Such a multimodal approach may refine disease staging, capture clinical heterogeneity, and identify complementary biomarkers for diagnosis and prognosis.

The current study aimed to compare fluid biomarker levels (NfL, GFAP, t‐tau, and derived ratios) between PSP patients and healthy controls and to examine associations between biomarkers and regional ^18^F‐PI‐2620 tau‐PET binding. We further evaluated the predictive value of plasma NfL/t‐tau ratios for clinical and cognitive outcomes and tested whether adding tau‐PET DVR or MRI‐derived brain volumes improved prediction beyond plasma biomarkers. We hypothesized that (1) biomarker ratios would be elevated in PSP compared with controls, (2) higher ratios would be associated with increased subcortical tau‐PET binding, (3) plasma NfL/t‐tau ratios would predict disease severity and processing speed, and integrating tau‐PET or MRI would provide complementary predictive value. These findings aim to improve understanding of PSP pathophysiology and highlight the potential of combining fluid and imaging biomarkers to enhance diagnosis, prognosis, and clinical trial design.

## 2 Methods

### 2.1 Patient Enrolment and Sample Characteristics

Patients were recruited as part of the SEL003 clinical trial investigating sodium selenate for the treatment of PSP [27]. This cross‐sectional, multicentre analysis included the baseline data from 24 patients with clinically diagnosed probable PSP (Richardson's syndrome) collected across six clinical centres across Australia (Melbourne, Sydney, Brisbane, and Adelaide) between July 2021 to August 2025. Baseline assessments included ^18^F‐PI‐2620 tau‐PET, brain MRI, CSF and blood sampling, and cognitive testing. Additionally, CSF and blood samples from 11 age‐ and sex‐matched cognitively normal controls (CDR = 0, MMSE > 25, no known neurological disease) were included from the Monash University Brain and Cognitive Health (BACH) cohort. Diagnosis of PSP was based on the Movement Disorder Society criteria for probable PSP [7]. Demographic data and medical history were collected at screening. Informed written consent was obtained from all participants or their legal representatives, as well as a caregiver. Ethics approval was granted by the Alfred Health and Monash University Human Research Ethics Committees (local reference numbers 594/20, SEL003; HREC/69184/Alfred‐2020; BACH, 532/21).

### 2.2 CSF and Blood Analyses

Plasma and CSF samples were analyzed using the single molecule array (SIMOA) platform (Quanterix Corp., Lexington, MA) with the Neurology 4‐Plex A kits to measure NfL, GFAP, and t‐tau, following manufacturer protocols. All samples were collected in a fasting state, frozen at −80°C and analyzed by the same lab across two plates. Internal controls were run across both plates to confirm there were no effects of plate. Differences across plates were less than coefficients of variance across the dataset. Duplicate coefficients of variance [range] were: CSF GFAP = 5.02% [0.19%–53.3%]; plasma GFAP = 2.59% [0.07%–11.3%]; CSF NfL = 7.07% [0.23%–10.7%]; plasma NfL = 4.75% [1.1%–8.6%]; CSF tau = 2.72% [0.07%–8.6%]; plasma tau = 7.69% [0.03%–30.1%]. Derived biomarker ratios (NfL/t‐tau, GFAP/t‐tau, GFAP/NfL) were calculated.

### 2.3 MRI and PET Acquisition and Preprocessing

High‐resolution 3D T1‐weighted MRI scans were acquired on a 3 T system (0.8 mm isotropic voxels). ^18^F‐PI‐2620 tau‐PET scans (Life Molecular Imaging, Berlin, Germany) were acquired dynamically for 0–60 min post‐injection (185 MBq ± 10%), reconstructed into frames of 10 × 30 s, 5 × 60 s, and 10 × 300 s. Final PET voxel dimensions were 2.3 × 2.3 × 3.3 mm. Preprocessing was performed using FSL v6.0.7.10. PET images were first rigidly motion‐corrected, a Gaussian smoothing kernel (*σ* = 4 mm) was applied, and dynamic frames averaged. The images were linearly coregistered to the T1‐weighted MRI, followed by nonlinear normalization to MNI152 space.

Region of interests (ROI) were defined using the Brainnetome [28] atlas for cortical and subcortical regions (pallidum, putamen, caudate, accumbens, frontal, occipital, temporal, parietal, cingulate, and thalamus) and the Talairach [29] atlas for midbrain structures (red nucleus, subthalamic nucleus, substantia nigra). Regions within the basal ganglia (putamen, pallidum) were of particular interest due to their known tau deposition in PSP [4, 11, 12]. Reference regions included inferior cerebellar gray matter and bilateral temporal/orbital white matter, based on minimal off‐target ^18^F‐PI‐2620 [30].

Distribution volume ratio (DVR) maps were computed using the simplified reference tissue model 2 (SRTM2) in PMOD 4.1 (PMOD Technologies, Zurich, Switzerland). Preprocessed dynamic PET images were loaded into PMOD, together with time–activity curves extracted from the predefined reference regions in FSL. Parametric images of the non‐displaceable binding potential (BP_ND_) were generated and subsequently converted to DVR maps (DVR = BP_ND_ + 1). For comparison, standardized uptake value ratio (SUVr) images were calculated using the 20–40 min post‐injection window in FSL.

For volumetric analyses, T1‐weighted images were normalized, corrected for intracranial volume, and segmented. The frontal cortex was selected as the primary atrophy marker, based on our preliminary data and prior reports [31].

### 2.4 Cognitive and Clinical Assessment

Patients underwent a comprehensive cognitive and symptom assessment battery, including the PSP Rating Scale (PSPRS; global disease severity), Behavior Rating Inventory of Executive Function‐Adult (BRIEF, everyday executive functioning, ‘Never’ scale), Frontal Assessment Battery (FAB; frontal executive control), Digit Span subtest from the Wechsler Adult Intelligence Scale—Fourth Edition (working memory and attention; forwards and reverse trials), Trail Making Test (TMT A: processing speed; TMT B: set‐shifting/executive function), the Category Fluency Test (animals; semantic fluency), Controlled Oral Word Association Test (COWAT; phonemic fluency, using the letters ‘FAS’), Victoria Stroop Test (inhibitory control and cognitive flexibility), and the Hayling Sentence Completion Test (response inhibition and executive control).

### 2.5 Data Analysis

Statistical analyses were conducted using R (v4.3.1) and SPM12 (Matlab R2022a). A two‐sided significance threshold of *p* < 0.05 was applied. Demographic variables (age, sex) were compared between patients with PSP and healthy controls using Wilcoxon rank‐sum and chi‐squared tests, with effect sizes reported using rank‐biserial correlation or Cramér's *V*.

Biomarker levels (CSF and plasma) were compared between PSP and controls using Wilcoxon rank‐sum tests, with rank‐biserial effect sizes. ROC curve analyses evaluated diagnostic performance (pROC package). Optimal cut‐offs were determined using Youden's *J* statistic.

To determine the most suitable PI‐2620 quantification approach for subsequent analyses, we visually compared group‐averaged DVR and SUVr parametric images derived from both reference regions (inferior cerebellar gray matter and bilateral temporal/orbital white matter). Methods were compared using a priori criteria including contrast in PSP‐vulnerable regions and stability across reference regions. Given its larger size and more reliable quantification, particular emphasis was placed on the putamen. DVR showed higher spatial specificity and lower reference‐region dependence and was therefore selected as the primary PET outcome.

Associations between fluid biomarkers and regional tau‐PET binding were examined using linear mixed‐effects models (lme4 package). Separate models were fitted for each fluid biomarker (NfL, GFAP, t‐tau, their ratios) in both plasma and CSF. The dependent variable was tau binding; fixed effects were fluid biomarker, tau binding region, age, and sex. Random intercepts were specified for each participant to account for within‐subject variability. All continuous variables were *z*‐standardized within the PSP cohort (mean = 0, SD = 1) prior to modeling, allowing interpretation of standardized slopes (*β*). Scatterplots and regression lines illustrating these associations were generated using the raw, unstandardized values. Post hoc ROI‐specific slopes were estimated using *emmeans*, with FDR correction applied across ROIs. Voxel‐wise analyses confirmed spatial consistency of ROI findings in SPM12 with *k* > 100 voxels and *p* < 0.05 (FDR‐corrected).

We assessed the contributions of plasma biomarkers, tau‐PET, and MRI to clinical and cognitive outcomes, focusing on the NfL/t‐tau ratio, which was selected a priori for its strong discriminatory performance and consistent association with regional DVR. Associations with cognitive and clinical measures (PSPRS, BRIEF, FAB, TMT‐A and B, Digit Span forward and reverse, Stroop, FAS, Hayling, and Category Fluency) were examined using linear regression models adjusted for age and sex. Continuous variables used standardized slopes. To test whether imaging provided additional predictive value, we compared nested models including the biomarker alone, biomarker + tau‐PET DVR (putamen), and biomarker + frontal MRI volume. Improvements in model fit were assessed using partial *F*‐tests and Δ*R* ^2^. Standardized *β* coefficients with 95% CIs were summarized in a forest plot to illustrate the relative contributions of each modality.

## 3 Results

The PSP cohort consisted of 24 participants (median [IQR] age, 67 [63–71] years; 11 females [46%]). 11 controls (median [IQR] age, 65 [62–68] years; 7 females [64%]) were included. Patients and controls did not differ significantly in age and sex (Table 1). Clinical test results are provided in Table S1.

**TABLE 1:** Fluid biomarkers and demographics in controls and PSP.

		Controls (*n* = 11)	PSP (*n* = 24)	*r*_*rb*_ ^a^	*p*	AUC% (95% CI)	Opt. CO
		Mdn (IQR)	Mdn (IQR)				
Age	In years	65 (5.4)	67 (8.2)	0.15	0.38		
Sex, *n* (%)	Female	7 (64)	11 (46)	0.10^b^	0.54		
	Male	4 (36)	13 (54)				
Disease duration	In weeks	/	40 (162)				
NfL	CSF	506 (482)	1567 (1524)	0.6	< 0.001***	89 (75–100)	1004
	Plasma	15 (5.6)	20 (13)	0.4	0.02*	76 (58–93)	17.7
GFAP	CSF	8345 (5657)	16128 (6853)	0.49	0.005**	82 (62–100)	12950
	Plasma	120 (79.1)	170 (123)	0.22	0.2	64 (43–84)	155
t‐tau	CSF	143 (55)	109 (62)	0.21	0.3	64 (41–86)	135
	Plasma	3.6 (1.1)	3.1 (2.1)	0.23	0.2	64 (45–84)	2.1
NfL/t‐tau	CSF	5.8 (3.1)	15 (7)	0.76	< 0.001***	99 (97–100)	10.2
	Plasma	3.9 (1.2)	7.2 (8.9)	0.46	0.007**	80 (63–96)	5.1
GFAP/t‐tau	CSF	80 (27)	138 (74)	0.61	< 0.001***	89 (76–100)	104
	Plasma	43 (25)	65 (52)	0.41	0.02*	76 (58–94)	50
GFAP/NfL	CSF	14 (6.1)	9.8 (7.2)	0.41	0.02*	76 (58–95)	8.9
	Plasma	8.5 (4)	8 (5.2)	0.09	0.63	56 (35–76)	5.9

*Note:* Asterisks indicate significant Wilcoxon signed‐rank tests.

Abbreviations: CSF = cerebrospinal fluid; GFAP = glial fibrillary acidic protein; IQR = interquartile range; Mdn = median; NfL = neurofilament light chain; Opt. CO = optimal cut‐off, measured in pg/mL; PSP = progressive supranuclear palsy; t‐tau = total tau.

^a^ Effect size using rank‐biserial correlation.

^b^ Effect size using Cramér's *V* strength of association of the Chi‐square test.

* *p* < 0.05.

** *p* < 0.01.

*** *p* < 0.001.

### 3.1 Higher NfL, GFAP, and Ratio Levels in PSP Compared With Controls

**FIGURE 1:**
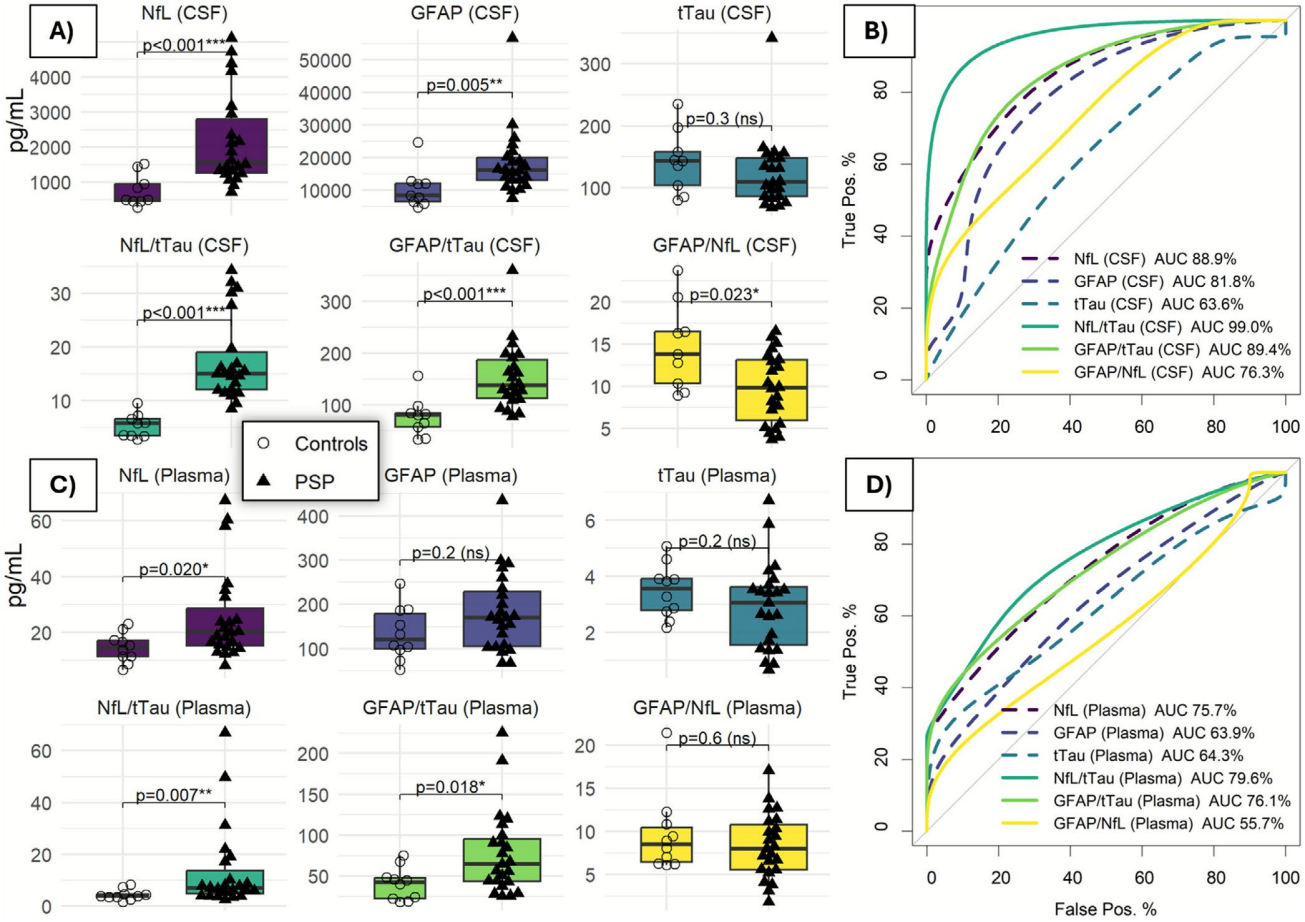
Fluid biomarkers in PSP vs controls. (A) Group comparisons for CSF levels and ratios. (B) ROC curves for CSF biomarkers and ratios. (C) Group comparisons for plasma levels and ratios. (D) ROC curves for plasma biomarkers and ratios. Vertical lines in boxplots indicate error bars; asteriks indicate significance (*p < 0.05; **p < 0.01, ***p < 0.001). Thicker ROC lines represent ratios; dashed lines represent individual fluid measures.

Patients with PSP displayed higher levels of NfL (CSF and plasma), GFAP (CSF), NfL/t‐tau (CSF and plasma), GFAP/t‐tau (CSF and plasma), and lower GFAP/NfL (CSF) compared with controls (Table 1; Figure 1). T‐tau levels were lower in PSP across both CSF and plasma but did not reach statistical significance. Intercorrelations between plasma and CSF biomarkers, including first‐order correlations of ratio measures, are reported in Table S2.

ROC analyses demonstrated strong discriminatory performance for CSF NfL (AUC = 0.89; 95% CI, 0.75–1.00; cut‐off = 1004 pg/mL), NfL/t‐tau (AUC = 0.99; 95% CI, 0.97–1.00; cut‐off = 10), and GFAP/t‐tau (AUC = 0.89; 95% CI, 0.76–1.00; cut‐off = 104). Plasma performance was lower overall, with NfL/t‐tau showing the highest discrimination (AUC = 0.80; 95% CI, 0.63–0.96; cut‐off = 5.1) (Table 1; Figure 1).

### 3.2 DVR With Inferior Cerebellar Gray Matter Reference Region Outperforms SUVr

**FIGURE 2:**
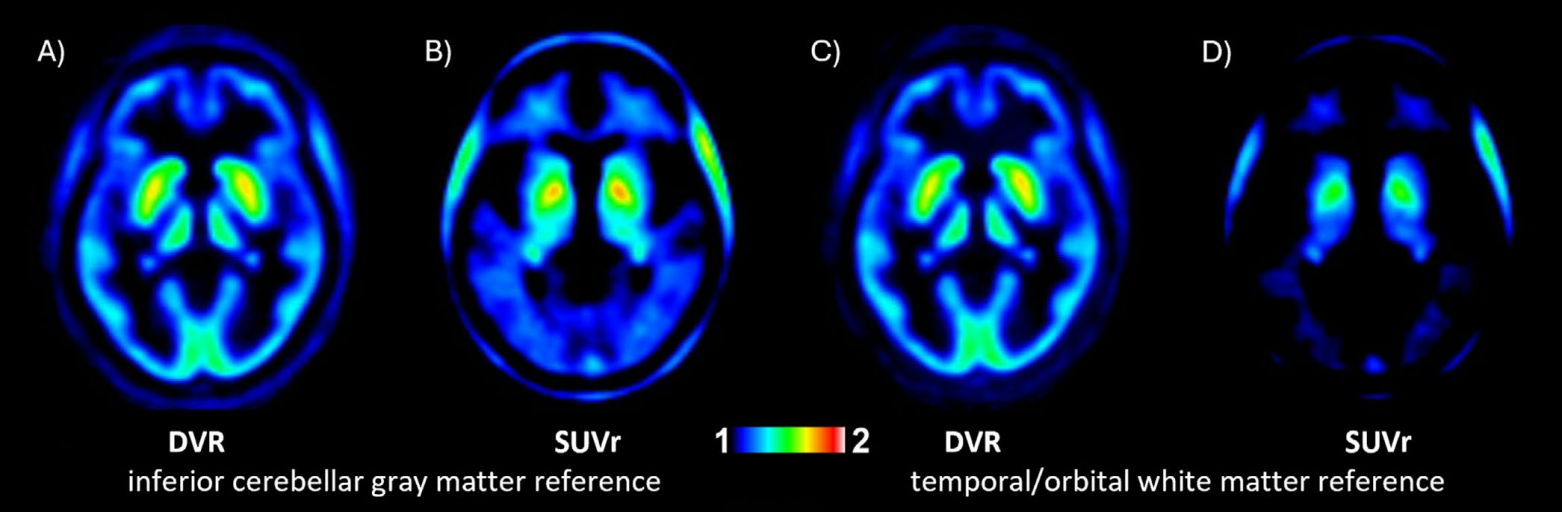
DVR vs SUVr across reference regions. Voxel‐wise PI‐2620 images averaged across patients comparing: (A) DVR, inferior cerebellar gray matter reference: Focal high uptake in the putamen with minimal signal in the pallidum. (B) SUVr, inferior cerebellar gray matter reference: Diffuse basal ganglia signal. (C) DVR, temporal/orbital white matter reference: Focal high uptake in the putamen with minimal signal in the pallidum. (D) SUVr, temporal/orbital white matter reference: Attenuated and diffuse basal ganglia signal. DVR = distribution volume ratio; SUVr = standardized uptake value ratio.

To select the most robust PI‐2620 PET quantification method for downstream analyses, we compared DVR and SUVr using two reference regions: inferior cerebellar gray matter and bilateral temporal/orbital white matter. Axial maps averaged across the patient cohort showed that DVR produced sharper, higher‐contrast binding localized to the putamen, with minimal signal in the pallidum. By contrast, SUVr images appeared more diffuse across the basal ganglia and were strongly dependent on the reference region, particularly attenuated when using temporal/orbital white matter. DVR maps were visually consistent across reference regions, demonstrating robustness. Based on these findings, DVR with inferior cerebellar gray matter was selected as the primary PET outcome (Figure 2).

### 3.3 Biomarker Ratios Are Associated With Subcortical Tau Uptake

**FIGURE 3:**
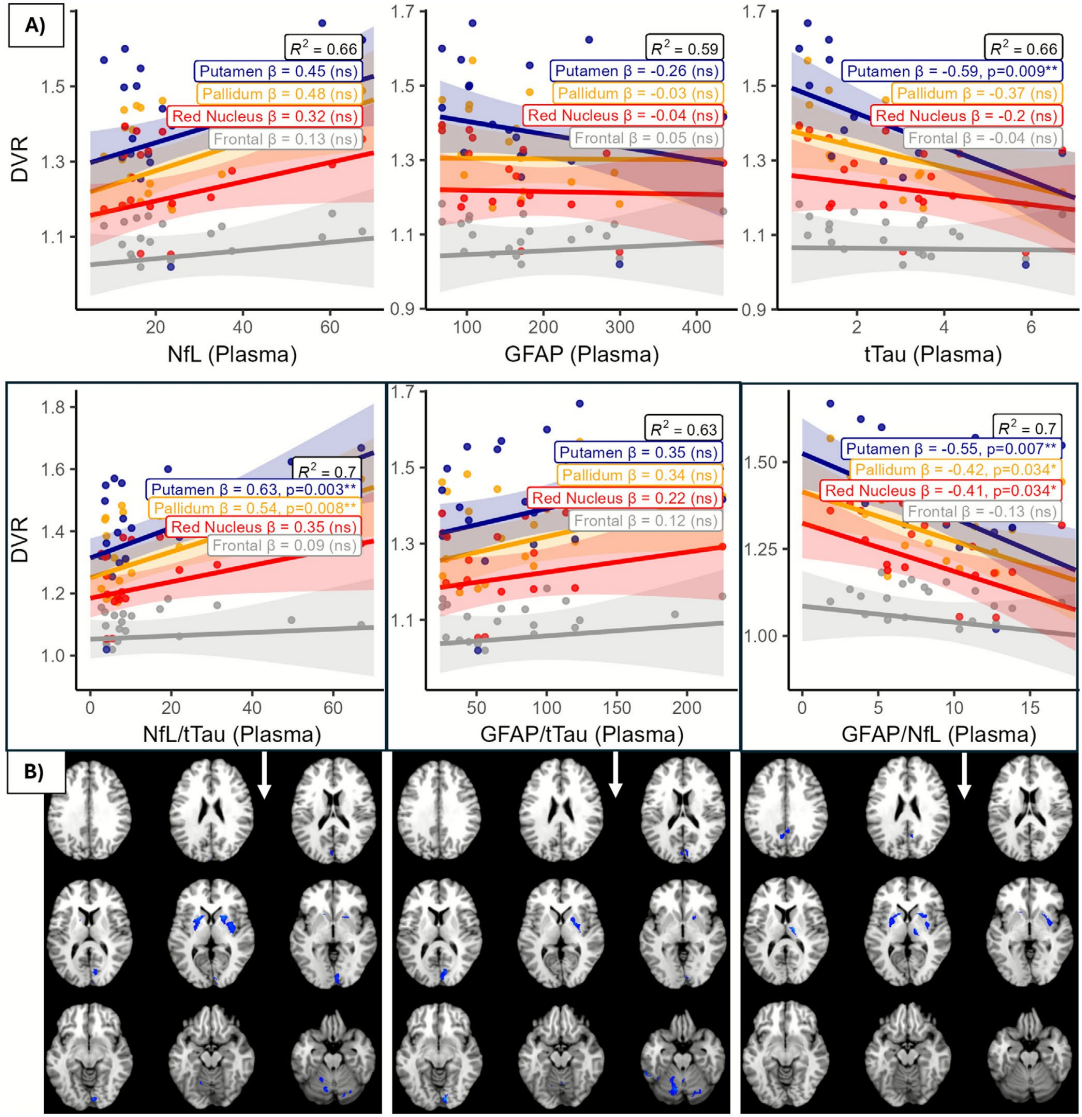
Regional associations of plasma biomarkers with DVR. (A) Standardized regression slopes (*β*) and their FDR‐corrected *p*‐value from ROI‐based mixed‐effects models (fixed effects: Fluid biomarker, tau‐binding region, age, sex); *p < 0.05; **p < 0.01; Biofluid concentrations are in pg/mL; shaded areas: 95% CIs. (B) Voxel‐wise multiple regression with plasma ratios as predictors (left: NfL/t‐tau; centre: GFAP/t‐tau; right: GFAP/NfL); threshold *p* < 0.05, uncorrected, age and sex as covariates; overlaid on T1 MRI template.

For ROI‐based analyses to assess associations between DVR and fluid biomarkers, we found significant plasma biomarker × region interactions. For t‐tau, higher levels were associated with lower DVR in the putamen (*β* = −0.59, 95% CI [−1.08–0.10]; *F*(12, 216) = 2.6, *p* = 0.003). For NfL/t‐tau, higher ratios were associated with higher DVR in the putamen (*β* = 0.63; 95% CI [0.15–1.11]) and pallidum (*β* = 0.54, 95% CI [0.07–1.02]; *F*(12, 216) = 3.5, *p* < 0.001). For GFAP/NfL, higher ratios were associated with lower DVR in the putamen (*β* = −0.55, 95% CI [−1.00–0.10]), pallidum (*β* = −0.42, 95% CI [−0.87–0.03]), and red nucleus (*β* = −0.41, 95% CI [−0.86–0.04]; *F*(12, 216) = 2.0, *p* = 0.02) (Figure 3A). Similar effects were seen for the CSF in those regions, although they did not reach significance due to larger variability. Detailed results for all ROI, biofluids, and CSF markers are shown Figure S1 and Table S3. Voxel‐wise analyses confirmed ROI findings, showing significant associations between plasma ratios (NfL/t‐tau, GFAP/t‐tau, GFAP/NfL) and tau uptake in the putamen (*p < 0*.05, *k* > 100), although effects did not survive FWE correction (Figure 3B).

### 3.4 Integrated Plasma and Imaging Markers Predict Clinical Outcomes

We next examined associations between plasma biomarkers and clinical measures, and whether tau‐PET DVR or MRI volume improved prediction beyond plasma biomarkers alone. Plasma NfL/t‐tau was selected a priori based on its strong discriminatory performance and consistent associations with DVR. Results for other plasma fluids (NfL, GFAP, t‐tau, and ratios) are shown in Table S4.

**FIGURE 4:**
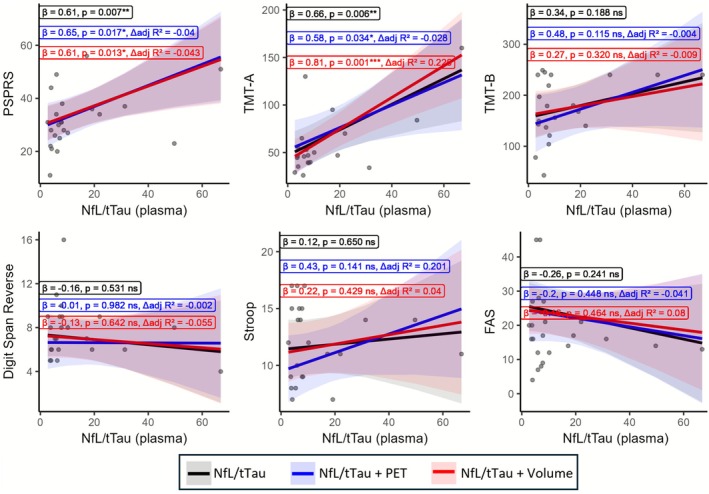
Added predictive value of PET and MRI beyond NfL/t‐tau for clinical outcomes. Associations between plasma NfL/t‐tau and clinical measures; black: NfL/t‐tau; red: NfL/t‐tau + PET DVR; blue: NfL/t‐tau + frontal MRI volume. DVR is measured in the putamen, MRI volume in the frontal region. Standardized regression slopes (*β*) and their *p*‐value from linear regression models, adjusted for age and sex; *p < 0.05; **p < 0.01, ***p < 0.001; Shaded areas: 95% CIs. FAS = Controlled Oral Word Association Test; NfL = neurofilament light chain; PSPRS = PSP Rating Scale; TMT‐A/B = Trail Making Test; tTau = total tau.

Linear regression models (adjusted for age and sex) revealed significant associations between higher NfL/t‐tau and increased disease severity scores (PSPRS, *β* = 0.61, 95% CI [0.19, 1.04], *p* = 0.007, *R* ^2^adj = 0.35), and slower processing speed (TMT‐A, *β* = 0.66, 95% CI [0.22, 1.10], *p* = 0.006, *R* ^2^adj = 0.33) (Figure 4). No significant associations were observed for BRIEF, FAB, TMT‐B, Stroop, Digit Span forward/reverse, FAS, Hayling Test, or the category fluency test.

To assess whether tau‐PET burden or MRI volume added predictive value, we compared nested models using Δ*R* ^2^ and partial F‐tests. We focused on the putamen for PET and the frontal region for MRI, as these have shown to have the most robust associations with NfL/t‐tau. For PSPRS, adding DVR (Δadj*R* ^2^ = −0.04, *p* = 0.8) or frontal volume (Δadj*R* ^2^ = −0.04, *p* = 0.9) did not improve model fit. For TMT‐A, DVR did not improve prediction (Δadj*R* ^2^ = −0.03, *p* = 0.5), but adding frontal volume significantly increased explained variance (Δadj*R* ^2^ = 0.2, *p* = 0.01) (Figure 4). No additional predictive value was observed for other clinical scales.

**FIGURE 5:**
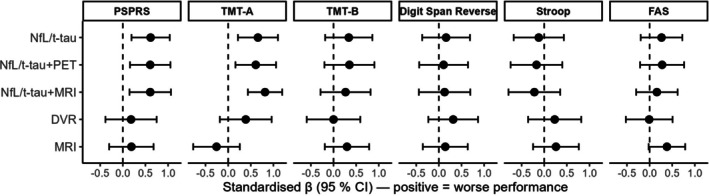
Standardized *β* coefficients (dots) and 95% confidence intervals (bars) for associations between plasma NfL/t‐tau and clinical/cognitive measures. Results are shown for individual models (NfL/t‐tau, PET DVR, MRI volume) and combined models (NfL/t‐tau + PET DVR, NfL/t‐tau + MRI volume). Note that for visualization purposes, test scores for the Digit Span Reverse, Stroop, FAS, and MRI volumes were multiplied by −1, so that all positive transformed *β* values indicate that predictor levels (low volume, high DVR, high NfL/t‐tau) are associated with worse performance. All models are adjusted for age and sex.

Finally, to directly compare the contributions of plasma NfL/t‐tau, DVR in the putamen, and frontal volume, we summarized the standardized *β* coefficients and 95% CIs across cognitive and clinical outcomes (Figure 5), from both individual and combined models. In combined models, coefficients represent the unique contribution of each predictor (NfL/t‐tau, DVR, MRI) to each clinical outcome after adjusting for the other modality. NfL/t‐tau emerged as the strongest independent predictor of disease severity (PSPRS). Frontal MRI volume contributed to processing speed (TMT‐A), and PET DVR provided minimal added value to clinical outcome.

## 4 Discussion

In this study we demonstrate that fluid biomarker ratios, particularly plasma NfL/t‐tau, are robust markers of pathological disease burden and clinical severity in PSP. Compared with healthy controls, patients with PSP exhibited elevated NfL and GFAP levels, higher NfL/t‐tau and GFAP/t‐tau ratios, and reduced t‐tau levels, in both CSF and plasma, consistent with previous reports. Plasma NfL/t‐tau was strongly associated with tau‐PET DVR in the putamen and pallidum, supporting its biological relevance. Consistent with previous research, PI‐2620 showed high sensitivity for detecting tau accumulation in the putamen and pallidum in PSP, aligning with post‐mortem tau distribution patterns [2, 4] and prior in vivo studies [11, 12, 32, 33, 34]. These findings confirm the biological validity of PI‐2620 for detecting PSP‐related tau pathology. Moreover, plasma NfL/t‐tau was the strongest independent predictor of disease severity and processing speed, while frontal MRI volume contributed modestly, and PI‐2620 tau‐PET DVR provided limited additional predictive value to cognitive performance. Taken together, these findings support the potential of fluid biomarker ratios as accessible and clinically meaningful tools for PSP phenotyping and disease monitoring, and that combining plasma with tau‐PET imaging may provide complementary insights into PSP pathophysiology.

Our results are consistent with prior studies demonstrating elevated plasma and CSF NfL in PSP relative to Parkinson's disease and controls [15, 16, 17, 35], supporting its role as a marker of neuroaxonal damage [36]. Baseline levels have previously been shown to predict disease severity [15, 18]. Although not specific to PSP, elevated NfL may reflect more aggressive neuronal loss in rapidly progressing conditions like PSP and frontotemporal dementia compared to Parkinson's disease or Alzheimer's disease [13, 14, 37].

Similarly, elevated GFAP levels align with evidence of astrocytic activation in PSP [13, 14, 17, 38]. Importantly, in the present study the associations were driven not by GFAP alone, but by GFAP relative to markers of tau‐related processes and axonal injury, as captured by the GFAP/t‐tau and GFAP/NfL ratios. Specifically, higher ratios were associated with lower tau‐PET binding in selected regions and better processing speed, despite absolute GFAP levels being higher in PSP than in controls. These findings suggest that the relative balance between astroglial activation, tau‐related pathology, and neuroaxonal injury changes across disease stages. Higher ratios may characterize earlier phases with relatively preserved axons and lower tau burden, while lower ratios may reflect later stages dominated by tau accumulation and neurodegeneration. However, the trajectory of these protein changes cannot be inferred from this cross‐sectional dataset and should be interpreted with caution. Longitudinal studies are therefore required to determine whether these ratios truly track stage‐specific shifts in PSP pathology.

T‐tau levels were reduced in patients with PSP relative to controls, consistent with previous reports of low t‐tau in PSP compared with other tauopathies [21, 22, 23]. CSF t‐tau has been associated with decreased perfusion in subcortical areas in PSP [12]. While t‐tau is often considered a non‐specific marker of neuronal injury [39, 40, 41, 42, 43, 44], PSP differs from Alzheimer's disease in both tau isoform composition and regional pathology, potentially explaining these lower levels. Our non‐significant results support the notion that t‐tau alone is insufficient as a PSP marker but becomes informative when interpreted within a ratio.

The results of this study demonstrate that fluid biomarker ratios (NfL/t‐tau, GFAP/t‐tau, GFAP/NfL) provide greater disease specificity and stronger associations with imaging and clinical measures than individual biomarkers. Plasma and CSF ratios outperformed NfL and GFAP alone in distinguishing PSP from controls, consistent with prior findings suggesting that ratios may better reflect tauopathy‐specific processes [26]. The markers may capture distinct temporal or regional patterns of neurodegeneration. It is possible that PSP predominantly affects subcortical myelinated axons, which have high NfL content but limited tau release, whereas cortical neurons, more affected in Alzheimer's disease, contribute proportionally greater tau. Alternatively, biomarker timing may differ, with NfL and GFAP increasing earlier in the disease course than t‐tau. Biologically, these ratios integrate markers of axonal injury (NfL) or astrocytic activation (GFAP) relative to neurodegeneration/aggregate tau burden (t‐tau), thereby enhancing signal‐to‐noise and reducing inter‐assay and inter‐individual scaling effects. Methodologically, ratios reduce redundancy between biomarkers, simplify statistical models, and provide more stable, interpretable measures, making them suitable for clinical use. Although our findings suggest that CSF measures more robustly distinguish PSP from controls compared to plasma measures, blood‐based biomarkers are less invasive, more accessible, and therefore a key focus of this study.

Despite biological relevance, tau‐PET DVR in the putamen and frontal MRI volume contributed minimally to explaining clinical variance beyond plasma NfL/t‐tau, with MRI improving prediction only for processing speed (TMT‐A). This does not imply redundancy but rather highlights that fluid biomarkers and imaging capture distinct, complementary aspects of PSP pathology. Plasma NfL likely reflects a global stage of neurodegeneration, indexing global downstream neuroaxonal injury and astroglial activation, which is closely tied to symptom severity, whereas tau‐PET measures regional tau deposition, which may not translate directly into functional impairment in a cross‐sectional analysis, particularly in such an early‐stage PSP cohort. This aligns with prior work showing weak or absent associations between tau‐PET and clinical severity in PSP [11].

Integrating these modalities therefore enables a more comprehensive and biologically grounded understanding of PSP progression and monitoring, and is particularly relevant for tau‐targeting disease‐modifying trials [27, 45]. Specifically, our findings suggest that plasma NfL/t‐tau ratios represent accessible, cost‐effective biomarkers for disease stage, downstream neurodegeneration, and may be useful for patient stratification and monitoring treatment response. In contrast, tau‐PET remains the most informative tool for in vivo detection of regional tau pathology, diagnostic confirmation, and may serve as a pharmacodynamic or target‐engagement endpoint, while MRI‐derived markers offer complementary information on structural degeneration and disease staging. Longitudinal studies will be essential to determine how these complementary markers track change over time.

This study has several limitations. First, the modest sample size, particularly of the control group, may limit generalizability and statistical power. Second, PSP diagnoses were not pathologically confirmed, although our homogeneous cohort of Richardson's syndrome cases likely reduces the risk of misclassification. Third, disease duration varied among participants, and only a few cases represented very early‐stage PSP. This heterogeneity may partly explain the strong biomarker differentiation observed between PSP and controls. The association of NfL/t‐tau with tau‐PET, disease severity, and cognition suggests that NfL may better differentiate groups at more advanced stages when neurodegeneration accelerates. It remains to be determined whether these biomarkers are equally sensitive in newly diagnosed or early‐stage patients. Future studies should compare total brain volume and PET measures with controls, adjust for disease stage, or incorporate longitudinal designs to clarify how fluid biomarker sensitivity evolves over time and detect pathological changes in the earliest stages, which represent an essential step toward enabling early intervention and improving clinical trial stratification. Finally, while NfL, GFAP, and t‐tau are informative, they are not PSP‐specific. Comparative studies including other neurodegenerative diseases, such as Parkinson's disease and atypical parkinsonism, are necessary to determine the specificity and discriminative value of these biomarkers. Fluid biomarkers lack regional specificity, underscoring the complementary role of imaging, and the consideration of newer markers such as MTBR‐tau_243_, p‐tau species, as well as emerging proteomic signatures that highlight dysregulated immune and axon‐guidance pathways in PSP [46, 47]. Future studies should also consider diurnal fluctuations in biomarker levels, as observed with Aβ in Alzheimer's disease [48]. In our study plasma was obtained in the morning, while CSF was mostly obtained after lunch.

This study demonstrates that fluid biomarker ratios, in particular plasma NfL/t‐tau, are robust, accessible markers of disease severity and cognitive dysfunction in PSP, outperforming tau‐PET and MRI for cross‐sectional prediction. While imaging measures provide valuable complementary insights into regional tau deposition and brain atrophy, their limited incremental contribution highlights the central role of fluid biomarkers for diagnosis, clinical monitoring, and trial design. Future multimodal, cross‐cohort, and longitudinal studies will be essential to define optimal strategies for combining fluid and imaging biomarkers to improve diagnosis, prognosis, and treatment evaluation in PSP.

## Author Contributions

Roxane Dilcher, Lucy Vivash, Charles B. Malpas, Kelly L. Bertram, Craig Despott, Terence J. O'Brien contributed to the conception and design of the study, and interpretation of results. Roxane Dilcher, Lucy Vivash, Craig Despott, William T. O'Brien contributed to the acquisition and analysis of data. Roxane Dilcher contributed to the statistical analysis and drafting of the manuscript. Lucy Vivash, Charles B. Malpas, William T. O'Brien, Stuart J. McDonald, Kelly L. Bertram, Craig Despott, Lukas Frontzkowski, Matthew P. Pase, Meng Law, Terence J. O'Brien contributed to the critical review of the manuscript for important intellectual content. William T. O'Brien, Stuart J. McDonald, Craig Despott, Kelly L. Bertram, Matthew P. Pase, Lukas Frontzkowski, Meng Law, Terence J. O'Brien, Lucy Vivash contributed to the administrative, technical, or material support. All authors approve and agree to be accountable for all aspects of the work in ensuring that questions related to the accuracy or integrity of any part of the work are appropriately investigated and resolved.

## Funding

This work was supported by Brain Foundation, Monash University, National Health and Medical Research Council (APP1176426, GTN1158384, GTN2009264), Alzheimer’s Association (AARG‐NTF‐22‐971405), Medical Research Future Fund (MRF1200254).

## Conflicts of Interest

The authors declare no conflicts of interest.

## Supporting information


**Data S1:** acn370327‐sup‐0001‐supinfo.docx.

## Data Availability

The data that support the findings will be available by contacting the corresponding author. Data will not be immediately available to allow for commercialization of research findings.
