# Supplementary material for: Fluid Biomarkers of Disease Burden and Cognitive Dysfunction in Progressive Supranuclear Palsy
Source: Ann Clin Transl Neurol. 2026 Jan 28;13(7):1422–32. doi: 10.1002/acn3.70327 (PMC13358566; doi:10.1002/acn3.70327)
Supplement: Supplementary file 1 — Data S1: acn370327‐sup‐0001‐supinfo.docx. [file ACN3-13-1422-s001.docx]

**Supplemental material**

**Table S1.** Average test results, DVR (putamen), and volume (frontal) in the PSP cohort

| **Test/imaging modality** | **N** | **Mdn (IQR)** |
| --- | --- | --- |
| PSPRS | 21 | 31.0 (11.0) |
| BRIEF | 21 | 35.0 (25.0) |
| FAB | 21 | 14.0 (3.0) |
| TMT-A | 20 | 48.5 (34.2) |
| TMT-B | 20 | 179.5 (97.8) |
| Digit Span Forward | 21 | 10.0 (3.0) |
| Digit Span Reverse | 21 | 7.0 (2.0) |
| Stroop | 21 | 12.0 (6.0) |
| FAS | 21 | 16.0 (8.0) |
| Hayling | 21 | 4.0 (4.0) |
| Category Fluency | 21 | 12.0 (5.0) |
| Putamen DVR (Mean, SD) | 21 | 1.442 (0.168) |
| Frontal volume (Mean, SD) | 21 | 0.888 (0.234) |

PSPRS= PSP Rating Scale, TMT=Trail Making Test, COWAT=Controlled Oral Word Association Test.

**Table S2:** Spearman correlation matrix of plasma and CSF biomarkers in the PSP cohort.

|  | NfL (CSF) | NfL (Plasma) | NfL/tTau (CSF) | NfL/tTau (Plasma) | GFAP (CSF) | GFAP (Plasma) | GFAP/tTau (CSF) | GFAP/tTau (Plasma) | Tau (CSF) | Tau (Plasma) | GFAP/NfL (CSF) | GFAP/NfL (Plasma) |
| --- | --- | --- | --- | --- | --- | --- | --- | --- | --- | --- | --- | --- |
| NfL (CSF) | 1 (p=NA) | 0.69 (p=0.001) | 0.68 (p=0) | 0.57 (p=0.007) | 0.48 (p=0.023) | 0.32 (p=0.154) | -0.16 (p=0.484) | 0.33 (p=0.139) | 0.72 (p=0) | -0.11 (p=0.626) | -0.66 (p=0.001) | -0.45 (p=0.042) |
| NfL (Plasma) | 0.69 (p=0.001) | 1 (p=NA) | 0.49 (p=0.025) | 0.53 (p=0.009) | 0.27 (p=0.234) | 0.52 (p=0.011) | -0.24 (p=0.286) | 0.26 (p=0.226) | 0.51 (p=0.017) | 0.02 (p=0.932) | -0.65 (p=0.001) | -0.49 (p=0.017) |
| NfL/tTau (CSF) | 0.68 (p=0) | 0.49 (p=0.025) | 1 (p=NA) | 0.46 (p=0.034) | 0.12 (p=0.584) | 0.12 (p=0.59) | 0.21 (p=0.344) | 0.2 (p=0.388) | 0.05 (p=0.813) | -0.11 (p=0.626) | -0.58 (p=0.005) | -0.48 (p=0.029) |
| NfL/tTau (Plasma) | 0.57 (p=0.007) | 0.53 (p=0.009) | 0.46 (p=0.034) | 1 (p=NA) | 0.18 (p=0.447) | 0.01 (p=0.975) | -0.25 (p=0.278) | 0.75 (p=0) | 0.36 (p=0.112) | -0.77 (p=0) | -0.5 (p=0.022) | -0.62 (p=0.002) |
| GFAP (CSF) | 0.48 (p=0.023) | 0.27 (p=0.234) | 0.12 (p=0.584) | 0.18 (p=0.447) | 1 (p=NA) | 0.56 (p=0.008) | 0.45 (p=0.036) | 0.49 (p=0.023) | 0.61 (p=0.003) | 0.03 (p=0.902) | 0.26 (p=0.244) | 0.28 (p=0.227) |
| GFAP (Plasma) | 0.32 (p=0.154) | 0.52 (p=0.011) | 0.12 (p=0.59) | 0.01 (p=0.975) | 0.56 (p=0.008) | 1 (p=NA) | 0.32 (p=0.154) | 0.28 (p=0.188) | 0.32 (p=0.153) | 0.38 (p=0.071) | 0.08 (p=0.733) | 0.41 (p=0.05) |
| GFAP/tTau (CSF) | -0.16 (p=0.484) | -0.24 (p=0.286) | 0.21 (p=0.344) | -0.25 (p=0.278) | 0.45 (p=0.036) | 0.32 (p=0.154) | 1 (p=NA) | 0.11 (p=0.638) | -0.34 (p=0.126) | 0.22 (p=0.33) | 0.63 (p=0.002) | 0.53 (p=0.014) |
| GFAP/tTau (Plasma) | 0.33 (p=0.139) | 0.26 (p=0.226) | 0.2 (p=0.388) | 0.75 (p=0) | 0.49 (p=0.023) | 0.28 (p=0.188) | 0.11 (p=0.638) | 1 (p=NA) | 0.36 (p=0.108) | -0.72 (p=0) | 0 (p=1) | -0.07 (p=0.75) |
| Tau (CSF) | 0.72 (p=0) | 0.51 (p=0.017) | 0.05 (p=0.813) | 0.36 (p=0.112) | 0.61 (p=0.003) | 0.32 (p=0.153) | -0.34 (p=0.126) | 0.36 (p=0.108) | 1 (p=NA) | -0.09 (p=0.687) | -0.29 (p=0.198) | -0.19 (p=0.401) |
| Tau (Plasma) | -0.11 (p=0.626) | 0.02 (p=0.932) | -0.11 (p=0.626) | -0.77 (p=0) | 0.03 (p=0.902) | 0.38 (p=0.071) | 0.22 (p=0.33) | -0.72 (p=0) | -0.09 (p=0.687) | 1 (p=NA) | 0.17 (p=0.471) | 0.41 (p=0.051) |
| GFAP/NfL (CSF) | -0.66 (p=0.001) | -0.65 (p=0.001) | -0.58 (p=0.005) | -0.5 (p=0.022) | 0.26 (p=0.244) | 0.08 (p=0.733) | 0.63 (p=0.002) | 0 (p=1) | -0.29 (p=0.198) | 0.17 (p=0.471) | 1 (p=NA) | 0.78 (p=0) |
| GFAP/NfL (Plasma) | -0.45 (p=0.042) | -0.49 (p=0.017) | -0.48 (p=0.029) | -0.62 (p=0.002) | 0.28 (p=0.227) | 0.41 (p=0.05) | 0.53 (p=0.014) | -0.07 (p=0.75) | -0.19 (p=0.401) | 0.41 (p=0.051) | 0.78 (p=0) | 1 (p=NA) |

NfL=neurofilament light chain, GFAP=glial fibrillary acidic protein, tTau=total tau

**Table S3:** Relationships between tau uptake and fluid biomarkers (pg/mL) across brain regions

|  |  | **CSF** | | | **Plasma** | | |
| --- | --- | --- | --- | --- | --- | --- | --- |
|  |  | **Estimate (β)** | **(95 % CI)** | | **Estimate (β)** | **(95 % CI)** | |
| **NfL** | ACC | 0.30 | [-0.26 | 0.87] | 0.11 | [-0.40 | 0.63] |
|  | Accumbens | 0.38 | [-0.19 | 0.95] | 0.20 | [-0.31 | 0.71] |
|  | Caudate | 0.42 | [-0.15 | 0.98] | 0.23 | [-0.28 | 0.75] |
|  | Frontal | 0.29 | [-0.28 | 0.86] | 0.13 | [-0.38 | 0.64] |
|  | Occipital | 0.44 | [-0.13 | 1.01] | 0.28 | [-0.23 | 0.79] |
|  | Pallidum | 0.69** | [0.12 | 1.26] | 0.48 | [-0.03 | 0.99] |
|  | Post. Cingulate | 0.43 | [-0.14 | 0.99] | 0.28 | [-0.23 | 0.79] |
|  | Putamen | 0.65** | [0.08 | 1.21] | 0.45 | [-0.06 | 0.96] |
|  | Red Nucleus | 0.53* | [-0.04 | 1.09] | 0.32 | [-0.19 | 0.83] |
|  | Substantia Nigra | 0.38 | [-0.18 | 0.95] | 0.23 | [-0.28 | 0.74] |
|  | Subthal. Nucleus | 0.40 | [-0.17 | 0.97] | 0.24 | [-0.27 | 0.75] |
|  | Temporal | 0.35 | [-0.21 | 0.92] | 0.17 | [-0.34 | 0.68] |
|  | Thalamus | 0.30 | [-0.26 | 0.87] | 0.05 | [-0.46 | 0.56] |
| **GFAP** | ACC | 0.15 | [-0.47 | 0.78] | 0.12 | [-0.40 | 0.65] |
|  | Accumbens | 0.10 | [-0.52 | 0.73] | -0.07 | [-0.60 | 0.46] |
|  | Caudate | 0.11 | [-0.52 | 0.73] | -0.12 | [-0.65 | 0.41] |
|  | Frontal | 0.18 | [-0.45 | 0.80] | 0.05 | [-0.47 | 0.58] |
|  | Occipital | 0.25 | [-0.38 | 0.88] | 0.11 | [-0.42 | 0.64] |
|  | Pallidum | 0.38 | [-0.25 | 1.01] | -0.03 | [-0.55 | 0.50] |
|  | Post. Cingulate | 0.08 | [-0.55 | 0.70] | 0.09 | [-0.44 | 0.62] |
|  | Putamen | 0.02 | [-0.61 | 0.65] | -0.26 | [-0.79 | 0.27] |
|  | Red Nucleus | 0.19 | [-0.43 | 0.82] | -0.04 | [-0.57 | 0.49] |
|  | Substantia Nigra | 0.13 | [-0.50 | 0.75] | -0.09 | [-0.62 | 0.44] |
|  | Subthal. Nucleus | 0.10 | [-0.53 | 0.72] | -0.10 | [-0.63 | 0.42] |
|  | Temporal | 0.24 | [-0.39 | 0.86] | 0.04 | [-0.49 | 0.57] |
|  | Thalamus | 0.15 | [-0.47 | 0.78] | -0.15 | [-0.68 | 0.38] |
| **tTau** | ACC | 0.51** | [0.05 | 0.97] | -0.07 | [-0.56 | 0.42] |
|  | Accumbens | 0.47** | [0.01 | 0.93] | -0.15 | [-0.64 | 0.34] |
|  | Caudate | 0.45** | [-0.01 | 0.91] | -0.19 | [-0.68 | 0.30] |
|  | Frontal | 0.42** | [-0.03 | 0.88] | -0.04 | [-0.53 | 0.45] |
|  | Occipital | 0.60** | [0.14 | 1.06] | -0.09 | [-0.58 | 0.40] |
|  | Pallidum | 0.67** | [0.22 | 1.13] | -0.37 | [-0.85 | 0.12] |
|  | Post. Cingulate | 0.57** | [0.11 | 1.03] | -0.26 | [-0.75 | 0.23] |
|  | Putamen | 0.54** | [0.08 | 0.99] | -0.59** | [-1.08 | -0.10] |
|  | Red Nucleus | 0.61** | [0.16 | 1.07] | -0.20 | [-0.69 | 0.29] |
|  | Substantia Nigra | 0.44** | [-0.02 | 0.90] | -0.19 | [-0.68 | 0.29] |
|  | Subthal. Nucleus | 0.49** | [0.04 | 0.95] | -0.19 | [-0.68 | 0.30] |
|  | Temporal | 0.50** | [0.04 | 0.96] | -0.04 | [-0.53 | 0.45] |
|  | Thalamus | 0.53** | [0.07 | 0.98] | -0.11 | [-0.60 | 0.38] |
| **NfL/tTau** | ACC | -0.02 | [-0.74 | 0.69] | 0.04 | [-0.44 | 0.52] |
|  | Accumbens | 0.04 | [-0.68 | 0.75] | 0.23 | [-0.25 | 0.71] |
|  | Caudate | 0.10 | [-0.61 | 0.82] | 0.27 | [-0.21 | 0.74] |
|  | Frontal | 0.00 | [-0.72 | 0.72] | 0.09 | [-0.39 | 0.56] |
|  | Occipital | 0.03 | [-0.68 | 0.75] | 0.23 | [-0.25 | 0.71] |
|  | Pallidum | 0.26 | [-0.46 | 0.98] | 0.54** | [0.07 | 1.02] |
|  | Post. Cingulate | 0.06 | [-0.66 | 0.78] | 0.22 | [-0.26 | 0.70] |
|  | Putamen | 0.30 | [-0.42 | 1.02] | 0.63** | [0.15 | 1.11] |
|  | Red Nucleus | 0.11 | [-0.61 | 0.83] | 0.35 | [-0.13 | 0.82] |
|  | Substantia Nigra | 0.06 | [-0.65 | 0.78] | 0.27 | [-0.21 | 0.74] |
|  | Subthal. Nucleus | 0.03 | [-0.69 | 0.75] | 0.27 | [-0.20 | 0.75] |
|  | Temporal | 0.00 | [-0.71 | 0.72] | 0.14 | [-0.34 | 0.61] |
|  | Thalamus | -0.09 | [-0.81 | 0.63] | 0.10 | [-0.38 | 0.58] |
| **GFAP/tTau** | ACC | -0.38 | [-0.92 | 0.16] | 0.14 | [-0.35 | 0.62] |
|  | Accumbens | -0.42 | [-0.96 | 0.12] | 0.11 | [-0.37 | 0.60] |
|  | Caudate | -0.36 | [-0.90 | 0.18] | 0.11 | [-0.38 | 0.59] |
|  | Frontal | -0.26 | [-0.80 | 0.28] | 0.12 | [-0.37 | 0.60] |
|  | Occipital | -0.41 | [-0.95 | 0.13] | 0.19 | [-0.29 | 0.67] |
|  | Pallidum | -0.32 | [-0.86 | 0.22] | 0.34 | [-0.14 | 0.83] |
|  | Post. Cingulate | -0.55* | [-1.09 | -0.01] | 0.28 | [-0.20 | 0.76] |
|  | Putamen | -0.56* | [-1.10 | -0.02] | 0.35 | [-0.13 | 0.84] |
|  | Red Nucleus | -0.48* | [-1.02 | 0.06] | 0.22 | [-0.26 | 0.70] |
|  | Substantia Nigra | -0.34 | [-0.88 | 0.20] | 0.14 | [-0.35 | 0.62] |
|  | Subthal. Nucleus | -0.44 | [-0.98 | 0.10] | 0.15 | [-0.33 | 0.64] |
|  | Temporal | -0.30 | [-0.84 | 0.24] | 0.10 | [-0.38 | 0.59] |
|  | Thalamus | -0.40 | [-0.94 | 0.14] | 0.00 | [-0.49 | 0.48] |
| **GFAP/NfL** | ACC | -0.28 | [-0.91 | 0.36] | -0.10 | [-0.55 | 0.35] |
|  | Accumbens | -0.32 | [-0.95 | 0.31] | -0.27 | [-0.72 | 0.18] |
|  | Caudate | -0.34 | [-0.98 | 0.29] | -0.33 | [-0.78 | 0.12] |
|  | Frontal | -0.23 | [-0.86 | 0.40] | -0.13 | [-0.58 | 0.32] |
|  | Occipital | -0.32 | [-0.95 | 0.31] | -0.19 | [-0.64 | 0.26] |
|  | Pallidum | -0.40 | [-1.03 | 0.24] | -0.42* | [-0.87 | 0.03] |
|  | Post. Cingulate | -0.38 | [-1.02 | 0.25] | -0.27 | [-0.72 | 0.18] |
|  | Putamen | -0.51 | [-1.15 | 0.12] | -0.55** | [-1.00 | -0.10] |
|  | Red Nucleus | -0.44 | [-1.08 | 0.19] | -0.41* | [-0.86 | 0.04] |
|  | Substantia Nigra | -0.33 | [-0.96 | 0.30] | -0.34 | [-0.79 | 0.11] |
|  | Subthal. Nucleus | -0.37 | [-1.00 | 0.26] | -0.36 | [-0.81 | 0.09] |
|  | Temporal | -0.23 | [-0.87 | 0.40] | -0.13 | [-0.58 | 0.32] |
|  | Thalamus | -0.21 | [-0.84 | 0.42] | -0.19 | [-0.64 | 0.25] |

NfL=neurofilament light chain, GFAP=glial fibrillary acidic protein, tTau=total tau. Asterisks indicate significant effects on linear mixed effect models, with age and sex as fixed effects, **p*<0.05; ***p*<0.01, ****p*<0.001.

**Table S4: Associations between plasma fluids and clinical outcomes, and incremental predictive value of PET and MRI**

| **Test** | **Fluid** | | | **Fluid + PET** | | | **Fluid + MRI** | | |
| --- | --- | --- | --- | --- | --- | --- | --- | --- | --- |
|  | β | 95% CI | Adj. R² | ΔR² | β | 95% CI | ΔR² | β | 95% CI |
| **NfL (Plasma)** | | | | | | | | | |
| PSPRS | 0.41 | [-0.10, 0.93] | 0.12 | -0.04 | 0.35 | [-0.22, 0.93] | -0.05 | 0.38 | [-0.17, 0.94] |
| BRIEF | 0.08 | [-0.46, 0.62] | 0.03 | -0.03 | -0.01 | [-0.60, 0.59] | -0.06 | 0.08 | [-0.51, 0.66] |
| FAB | 0.14 | [-0.39, 0.66] | 0.08 | 0.02 | 0.01 | [-0.56, 0.58] | 0.01 | 0.22 | [-0.33, 0.76] |
| TMT-A | 0.36 | [-0.20, 0.92] | 0.01 | 0.06 | 0.21 | [-0.38, 0.80] | 0.1 | 0.48 | [-0.08, 1.04] |
| TMT-B | 0.33 | [-0.22, 0.88] | 0.06 | -0.04 | 0.4 | [-0.20, 1.01] | -0.01 | 0.26 | [-0.32, 0.83] |
| Digit Forward | 0.02 | [-0.58, 0.61] | -0.18 | -0.08 | 0.02 | [-0.65, 0.69] | 0.07 | 0.13 | [-0.47, 0.74] |
| Digit Reverse | -0.02 | [-0.58, 0.54] | -0.04 | 0.03 | 0.12 | [-0.49, 0.72] | -0.04 | 0.02 | [-0.58, 0.62] |
| Stroop | 0.45 | [-0.09, 0.98] | 0.05 | 0.3* | 0.71** | [0.22, 1.19] | 0.12 | 0.58* | [0.05, 1.10] |
| FAS | -0.39 | [-0.85, 0.08] | 0.29 | -0.04 | -0.35 | [-0.87, 0.17] | 0.06 | -0.29 | [-0.75, 0.17] |
| Hayling | 0.08 | [-0.46, 0.62] | 0.04 | -0.06 | 0.1 | [-0.51, 0.70] | -0.06 | 0.06 | [-0.52, 0.64] |
| Cat. Fluency | -0.29 | [-0.86, 0.28] | -0.08 | 0.01 | -0.41 | [-1.03, 0.21] | -0.07 | -0.31 | [-0.92, 0.31] |
| **GFAP (Plasma)** | | | | | | | | | |
| PSPRS | -0.09 | [-0.62, 0.45] | -0.03 | 0.01 | -0.02 | [-0.57, 0.53] | -0.01 | -0.13 | [-0.68, 0.42] |
| BRIEF | 0.03 | [-0.49, 0.55] | 0.02 | -0.01 | 0.08 | [-0.46, 0.62] | -0.06 | 0.03 | [-0.52, 0.57] |
| FAB | -0.15 | [-0.65, 0.35] | 0.09 | 0.02 | -0.08 | [-0.60, 0.43] | -0.02 | -0.12 | [-0.64, 0.40] |
| TMT-A | -0.39 | [-0.92, 0.14] | 0.05 | 0.08 | -0.3 | [-0.83, 0.22] | -0.02 | -0.35 | [-0.90, 0.19] |
| TMT-B | 0.17 | [-0.37, 0.72] | -0.01 | -0.07 | 0.17 | [-0.41, 0.76] | 0.02 | 0.12 | [-0.43, 0.67] |
| Digit Forward | -0.02 | [-0.59, 0.55] | -0.18 | -0.08 | -0.02 | [-0.63, 0.59] | 0.06 | 0.05 | [-0.52, 0.61] |
| Digit Reverse | 0.22 | [-0.30, 0.74] | 0.01 | 0 | 0.16 | [-0.38, 0.70] | -0.03 | 0.26 | [-0.29, 0.80] |
| Stroop | 0.56* | [0.08, 1.03] | 0.18 | 0.01 | 0.5* | [0.01, 0.99] | 0.11 | 0.63** | [0.18, 1.08] |
| FAS | -0.33 | [-0.78, 0.13] | 0.26 | 0.05 | -0.4 | [-0.85, 0.05] | 0.09 | -0.26 | [-0.69, 0.17] |
| Hayling | -0.03 | [-0.55, 0.48] | 0.03 | -0.06 | -0.03 | [-0.58, 0.52] | -0.05 | -0.05 | [-0.60, 0.49] |
| Cat. Fluency | -0.26 | [-0.80, 0.29] | -0.09 | -0.07 | -0.24 | [-0.82, 0.35] | -0.07 | -0.26 | [-0.84, 0.32] |
| **tTau (Plasma)** | | | | | | | | | |
| PSPRS | -0.41 | [-0.88, 0.07] | 0.14 | -0.05 | -0.36 | [-0.92, 0.21] | -0.03 | -0.39 | [-0.88, 0.09] |
| BRIEF | -0.15 | [-0.64, 0.35] | 0.05 | -0.04 | -0.06 | [-0.65, 0.52] | -0.06 | -0.15 | [-0.66, 0.37] |
| FAB | -0.09 | [-0.58, 0.40] | 0.07 | 0.04 | 0.08 | [-0.48, 0.63] | -0.01 | -0.11 | [-0.61, 0.39] |
| TMT-A | -0.43 | [-0.94, 0.07] | 0.09 | 0.02 | -0.29 | [-0.86, 0.28] | 0.05 | -0.46 | [-0.96, 0.04] |
| TMT-B | -0.28 | [-0.80, 0.24] | 0.04 | -0.03 | -0.38 | [-0.99, 0.22] | 0.03 | -0.26 | [-0.78, 0.26] |
| Digit Forward | -0.07 | [-0.62, 0.49] | -0.17 | -0.08 | -0.09 | [-0.75, 0.57] | 0.06 | -0.09 | [-0.64, 0.45] |
| Digit Reverse | -0.04 | [-0.56, 0.48] | -0.04 | 0.07 | -0.24 | [-0.82, 0.35] | -0.04 | -0.05 | [-0.59, 0.49] |
| Stroop | 0.17 | [-0.37, 0.70] | -0.11 | 0.04 | -0.01 | [-0.62, 0.60] | 0 | 0.15 | [-0.39, 0.69] |
| FAS | 0.06 | [-0.41, 0.53] | 0.16 | 0 | -0.06 | [-0.60, 0.48] | 0.12 | 0.03 | [-0.40, 0.47] |
| Hayling | 0.29 | [-0.19, 0.77] | 0.12 | -0.02 | 0.39 | [-0.17, 0.95] | -0.04 | 0.3 | [-0.19, 0.79] |
| Cat. Fluency | -0.18 | [-0.72, 0.36] | -0.12 | -0.07 | -0.15 | [-0.80, 0.49] | -0.07 | -0.19 | [-0.75, 0.38] |
| **NfL/tTau (Plasma)** | | | | | | | | | |
| PSPRS | 0.61** | [0.19, 1.04] | 0.35 | -0.04 | 0.65* | [0.13, 1.16] | -0.04 | 0.61* | [0.15, 1.06] |
| BRIEF | 0.07 | [-0.44, 0.59] | 0.03 | -0.02 | -0.05 | [-0.66, 0.57] | -0.06 | 0.07 | [-0.49, 0.63] |
| FAB | 0.06 | [-0.45, 0.57] | 0.07 | 0.05 | -0.14 | [-0.72, 0.44] | 0 | 0.13 | [-0.40, 0.66] |
| TMT-A | 0.66** | [0.22, 1.10] | 0.33 | -0.03 | 0.58* | [0.05, 1.11] | 0.23* | 0.81*** | [0.44, 1.19] |
| TMT-B | 0.34 | [-0.18, 0.86] | 0.07 | 0 | 0.48 | [-0.13, 1.09] | -0.01 | 0.27 | [-0.29, 0.82] |
| Digit Forward | -0.08 | [-0.65, 0.48] | -0.17 | -0.08 | -0.12 | [-0.81, 0.58] | 0.05 | 0.02 | [-0.56, 0.60] |
| Digit Reverse | -0.16 | [-0.69, 0.37] | -0.02 | 0 | -0.01 | [-0.63, 0.62] | -0.05 | -0.13 | [-0.69, 0.44] |
| Stroop | 0.12 | [-0.43, 0.68] | -0.12 | 0.2 | 0.43 | [-0.16, 1.03] | 0.04 | 0.22 | [-0.35, 0.79] |
| FAS | -0.26 | [-0.72, 0.20] | 0.23 | -0.04 | -0.2 | [-0.76, 0.36] | 0.08 | -0.16 | [-0.62, 0.30] |
| Hayling | 0.03 | [-0.48, 0.55] | 0.03 | -0.06 | 0.04 | [-0.59, 0.67] | -0.05 | 0 | [-0.55, 0.56] |
| Cat. Fluency | -0.12 | [-0.68, 0.44] | -0.14 | -0.01 | -0.27 | [-0.94, 0.39] | -0.08 | -0.13 | [-0.73, 0.48] |
| **GFAP/tTau (Plasma)** | | | | | | | | | |
| PSPRS | 0.31 | [-0.17, 0.78] | 0.07 | -0.03 | 0.25 | [-0.26, 0.76] | -0.04 | 0.28 | [-0.23, 0.78] |
| BRIEF | 0.08 | [-0.40, 0.57] | 0.03 | -0.03 | 0.02 | [-0.50, 0.55] | -0.06 | 0.08 | [-0.44, 0.60] |
| FAB | 0 | [-0.47, 0.48] | 0.06 | 0.05 | -0.1 | [-0.60, 0.39] | -0.01 | 0.06 | [-0.44, 0.55] |
| TMT-A | 0.15 | [-0.37, 0.68] | -0.08 | 0.12 | 0.03 | [-0.50, 0.55] | 0.04 | 0.24 | [-0.30, 0.78] |
| TMT-B | 0.41 | [-0.06, 0.87] | 0.15 | -0.03 | 0.46 | [-0.04, 0.96] | -0.01 | 0.35 | [-0.14, 0.84] |
| Digit Forward | 0 | [-0.53, 0.53] | -0.18 | -0.08 | 0 | [-0.58, 0.59] | 0.07 | 0.09 | [-0.45, 0.63] |
| Digit Reverse | 0.14 | [-0.36, 0.64] | -0.02 | 0.08 | 0.26 | [-0.25, 0.77] | -0.03 | 0.19 | [-0.33, 0.71] |
| Stroop | 0.28 | [-0.23, 0.78] | -0.05 | 0.19* | 0.44 | [-0.05, 0.92] | 0.07 | 0.36 | [-0.14, 0.87] |
| FAS | -0.28 | [-0.71, 0.15] | 0.24 | -0.03 | -0.24 | [-0.70, 0.23] | 0.08 | -0.2 | [-0.62, 0.22] |
| Hayling | -0.18 | [-0.66, 0.29] | 0.07 | -0.06 | -0.21 | [-0.73, 0.32] | -0.04 | -0.22 | [-0.72, 0.28] |
| Cat. Fluency | -0.13 | [-0.65, 0.40] | -0.14 | -0.03 | -0.19 | [-0.76, 0.37] | -0.08 | -0.13 | [-0.69, 0.43] |
| **GFAP/NfL (Plasma)** | | | | | | | | | |
| PSPRS | -0.38 | [-0.85, 0.08] | 0.12 | -0.05 | -0.34 | [-0.93, 0.25] | -0.03 | -0.37 | [-0.85, 0.11] |
| BRIEF | 0.04 | [-0.45, 0.53] | 0.02 | 0.02 | 0.23 | [-0.37, 0.83] | -0.06 | 0.04 | [-0.47, 0.56] |
| FAB | -0.29 | [-0.75, 0.16] | 0.16 | -0.03 | -0.19 | [-0.76, 0.38] | 0.01 | -0.31 | [-0.77, 0.15] |
| TMT-A | -0.47 | [-0.94, 0.00] | 0.14 | -0.01 | -0.34 | [-0.92, 0.24] | 0.06 | -0.5* | [-0.96, -0.04] |
| TMT-B | -0.13 | [-0.65, 0.39] | -0.02 | -0.05 | -0.21 | [-0.86, 0.44] | 0.03 | -0.1 | [-0.62, 0.42] |
| Digit Forward | 0.05 | [-0.49, 0.59] | -0.17 | -0.08 | 0.07 | [-0.62, 0.75] | 0.06 | 0.02 | [-0.51, 0.55] |
| Digit Reverse | 0.26 | [-0.23, 0.75] | 0.03 | -0.04 | 0.15 | [-0.46, 0.77] | -0.05 | 0.25 | [-0.25, 0.76] |
| Stroop | 0.12 | [-0.41, 0.65] | -0.12 | 0.06 | -0.11 | [-0.74, 0.52] | 0 | 0.1 | [-0.43, 0.63] |
| FAS | 0.08 | [-0.37, 0.54] | 0.16 | -0.01 | -0.05 | [-0.62, 0.51] | 0.12 | 0.05 | [-0.38, 0.48] |
| Hayling | -0.01 | [-0.50, 0.48] | 0.03 | -0.06 | 0 | [-0.63, 0.62] | -0.05 | 0 | [-0.51, 0.51] |
| Cat. Fluency | 0.12 | [-0.41, 0.65] | -0.14 | 0 | 0.29 | [-0.36, 0.95] | -0.08 | 0.12 | [-0.44, 0.67] |

NfL=neurofilament light chain, GFAP=glial fibrillary acidic protein, tTau=total tau, PSPRS=PSP Rating Scale, BRIEF=Behaviour Rating Inventory of Executive Function, FAB=Frontal Assessment Battery, TMT-A/B=Trail Making Test, FAS=Controlled Oral Word Association Test. Asterisks indicate significant effects, with age and sex as fixed effects, **p*<0.05; ***p*<0.01, ****p*<0.001.

**
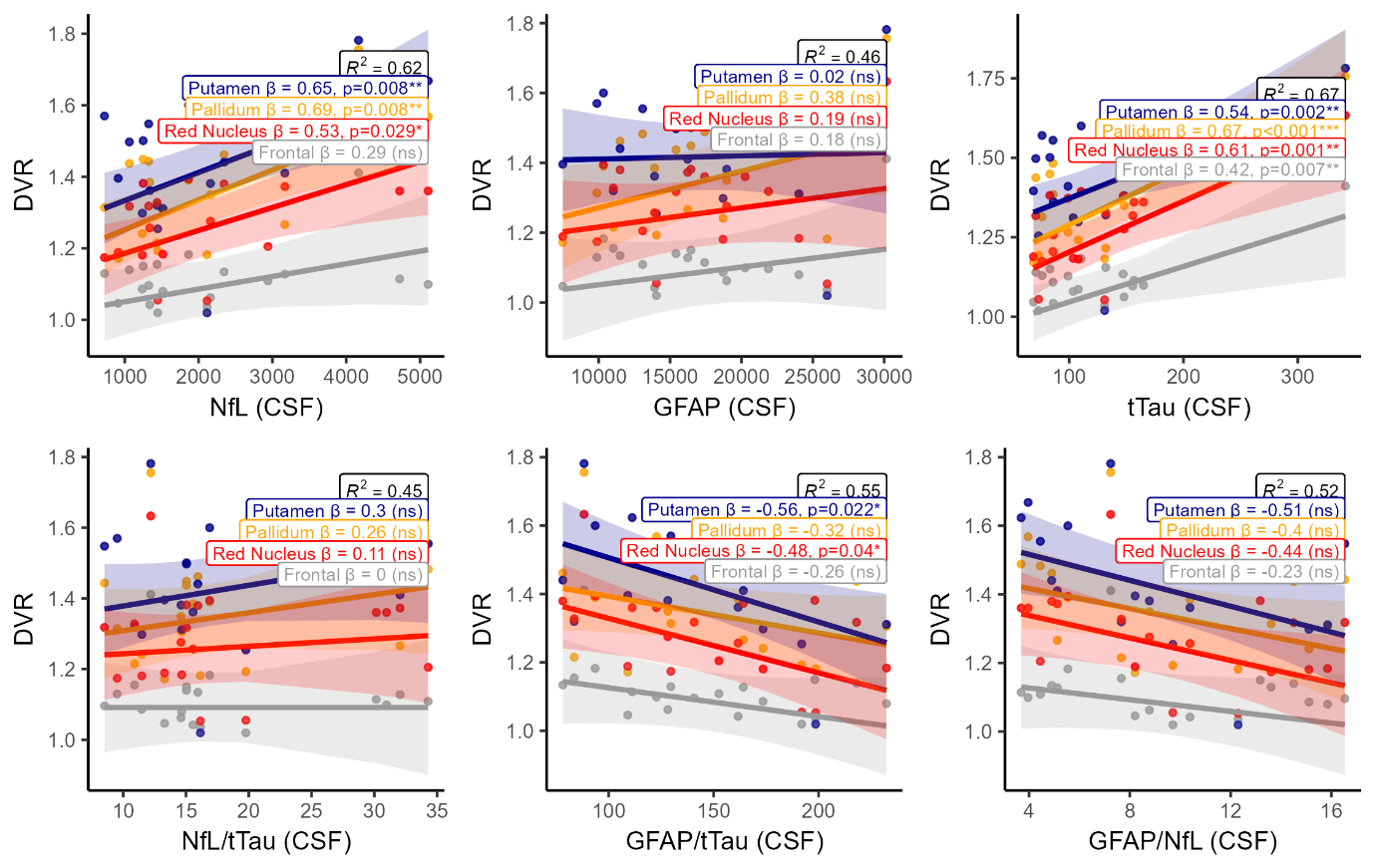
**

**Fig S1. Regional associations of CSF biomarkers with DVR.** A) Standardised regression slopes (β) and their FDR-corrected *p*-value from ROI-based mixed-effects models (fixed effects: fluid biomarker, tau-binding region, age, sex); **p*<.05; ***p*<.01, ****p*<.001; Biofluid concentrations are in pg/mL; shaded areas: 95% CIs.
